# Can SMEs benefit equally from supportive policies in China?

**DOI:** 10.1371/journal.pone.0280253

**Published:** 2023-03-30

**Authors:** Wenqi Zhang, Zuogong Wang

**Affiliations:** School of Economics, Henan University, Kaifeng, Henan, China; Hebei University of Economics and Business, CHINA

## Abstract

This paper applies quantitative and narrative approaches to fiscal and financial policies of Chinese small and medium enterprises (SMEs) in order to study the support effect of macro policies on micro enterprises. As the first researchers to focus on the firm heterogeneity of SMEs’ policy effects, we show that the “flood irrigation” supportive policies for SMEs have not achieved the expected “help the weaker” effect. Non-state-owned SMEs and small(micro) enterprises have a low sense of policy gain, which is contrary to some “positive” research conclusions from China. The mechanism study found that “ownership” and “scale” discrimination suffered by non-state-owned and small(micro) enterprises in the financing process are key. We suggest the supportive policies for SMEs should shift from “flood” to “precise drip” irrigation. The policy benefits of non-state-owned, small and micro enterprises need to be emphasized. More targeted policies need to be studied and provided. Our findings shed new light on the formulation of supportive policies for SMEs.

## Introduction

In today’s globalized, informationized, knowledge-based and market-oriented economy, small and medium-sized enterprises (SMEs), the most active factor in the social economy, play an irreplaceable role in the development of the national economy in both developed and developing countries. However, they are still in a disadvantaged position in the market competition, and face a poor external development environment, difficult and expensive financing and other difficulties, and so struggle to obtain a virtuous cycle of development. Countries have therefore made great efforts to provide support for SMEs and have shown their own policy measures. After years of practice, financial policies of SMEs [1–3]), tax incentives [4, 5] and subsidies [6, 7] and other fiscal policies have been proven to impact to different extents in various countries.

Fred et al.’s [8] investigation of SMEs in Nigeria, found the majority of interviewed SMEs operators believed the SMEs Development Agency of Nigeria (SMEDAN) facilitated access to finance. But many of those interviewed said they had experienced prejudice because they are not linked to any official in a SMEDAN office. Even if the loan is urgently needed, they cannot obtain the loan facility.

It seems that we should not be satisfied with overall “effectiveness”, as this is not a unique situation. In China, the initiation of a supportive government led policy has resulted in stronger enterprises always being preferred. Resources such as government subsidies, tax incentives and financial support are heavily tilted in favor of state-owned and large enterprises. Wang and Wang [9] found that state-owned enterprises (SOEs) receive more government subsidies and bank loans influenced by the government’s “helping hand”. Yao [10] examined the scale heterogeneity of private enterprises’ tax and fee reduction policy dividends among private enterprises and found large private enterprises enjoy more policy dividends than small and medium-sized counterparts. Meng et al. [11] pointed out that SOEs have a closer political connection with the government and can obtain financial subsidies more easily, but financial subsidies have been abused, resulting in excessive investment. Wang et al. [12] indicated that government subsidies tend to support large enterprise and play a more considerable role in it. Moreover, recent studies are exposing the policy preference to state-owned and large enterprises in strategic emerging industrial. Such as, green finance policies seem unable to benefit privately owned SMEs (e.g., [13–15], etc.). Green credits are likely to be available to state-owned and large enterprises that address financing constraints and risks rather than non-state-owned, small- and medium-sized enterprises.

In recent years, the Government has introduced and implemented a series of fiscal and financial policies and measures to relieve the difficulties of SMEs and support their development. In responding to the international financial crisis and COVID-19, the Government has offered an increasing amount of supportive policies to SMEs. This raises the question if the effect of SMEs’ fiscal and financial policies is similar to “support the stronger”. In addition, there are also questions about the policy gain of non-state-owned SMEs and small (micro) enterprises and if they can be treated equally favorably by fiscal and financial support policies?

But after the introduction of the domestic and international “double cycle” strategy and the establishment of the Beijing Stock Exchange, SMEs obtained a new opportunity for rapid development. And this leads us to think about how effective the implementation of fiscal and financial policy measures for SMEs have been. In addition, we are also led to ask how much interventions have helped the future growth of SMEs and to ask about the way out for non-state-owned SMEs and small (micro) enterprises.

In order to answer these questions, this paper first develops quantitative guidelines for fiscal and financial policies. It then constructs a fiscal and financial policy index for SMEs. In focusing on the heterogeneity of the property rights nature and enterprises size of, it investigates the micro effects and conduction mechanism of SMEs’ fiscal and financial policies by using enterprises’ investment efficiency, which is directly related to their future growth and value performance.

Our findings contribute to existing research in at least three ways. First, this paper focuses on the firm heterogeneity of SMEs’ policy effects. Most existing studies of SMEs’ policy effects are only focus on the whole. But the strength of the policy effects of non-state-owned /state-owned and large/small companies are indispensable when examining the effect of policies and should receive extra attention from policy makers. Second, this paper isa new attempt to measure macroeconomic policies. Existing research of the measurement of macroeconomic policies consists of questionnaire surveys that have been distributed to enterprises (e.g., [16, 17]). Although these surveys can obtain unique data, their authenticity is open to question and they have a small sample size. Moreover, the policy proxy variables that most scholars use operate at the level of enterprise data–for example, fiscal policy proxies are government subsidies, ratio of government subsidies to operating income, dummy variables of government subsidies (e.g., [18, 19], etc.). The use of enterprise-level data examines the effect of policies that have been implemented and measures the preferential policy enterprises have received. However, this does not reflect the strength and trend of macro policies, does not really establish the link between the macro-level supportive policies and the micro-level SMEs, and cannot tell how fiscal and financial policies are transmitted to SMEs.

In taking this into account, we use quantitative and narrative approaches to identify and construct a fiscal and financial policy index for SMEs that is based on SMEs’ supportive policies this appears to be a first in the policy research of SMEs in China. Third, this study also systematically analyzes the impact of macro fiscal and financial policies on the investment efficiency of micro enterprises, and it does this by applying the two conduction mechanisms of investment opportunities and financing constraints. And the analysis of firm heterogeneity is included in the conduction mechanisms. This paper could help to improve the policy effect of SMEs and promote their healthy and sustainable development.

The remainder of the paper follows. Section 2 develops the paper’s hypotheses; Section 3 constructs the fiscal and financial policy index of SMEs and offers a brief analysis; Section 4 discusses the data, variables and estimation models; Section 5 presents and discusses the results; Section 6 provides further analysis and conducts robustness tests; Section 7 presents the paper’s conclusions.

## Theoretical analysis and research hypothesis

This paper attempts to measure the implementation effect of SMEs’ fiscal and financial policies at the micro level of enterprises and analyze the impact of fiscal and financial policies on SMEs’ investment efficiency. In order to further analyze the mechanism, the rule of Capital Follows Profitability followed by enterprises and government’s fiscal and financial policy instruments are need to be discussed together. According to the Capital Follows Profitability economic rule of enterprise investment decision, the realization of enterprise efficiency investment depends on the following constraints: one is investment opportunity and the other is financing capacity (see [20–22]).

If an enterprise does not have a financing constraint, then an enterprise’s investment expenditures depend on the investment opportunities it faces [22]. We argue that SMEs’ fiscal and financial policies can increase their investment opportunities, and suggests this can in turn have an incentive effect for investment efficiency. In terms of the macroeconomic environment, the introduction of an active fiscal policy can promote private investment scale, drive social consumption and increase export potential through demand-side stimulation [23]. On the other hand, when supporting SMEs, the government will give tax incentives, set up special funds, and also provide support to SMEs in the form of land and plants, to take two examples. In this way, the tax burden of SMEs, the cost of fixed asset investment and the investment cost of enterprises will fall. Moreover, the government will also introduce supportive fiscal policies for SMEs in specific industries with the aim of encouraging structural adjustment and independent innovation; and enterprises will adjust their investment behavior accordingly in order to obtain tax concessions and government subsidies. Meanwhile, within the supported industries, government procurement and investment will cause market demand within the industries to increase, and the investment income of SMEs in the industries will also increase. Financial policies will tilt bank credit to SMEs, and commercial banks will prefer industries, enterprises with good development prospects and high profitability or investment projects when allocating loan resources, and this will force enterprises to strive to seize good investment opportunities and constantly seek progress and transformation when seeking to improve their investment efficiency and profitability [24].

However, the existence of the “double dual structure” of China’s economic and financial systems “has led to a serious imbalance and inequity in the allocation of financial resources in terms of opportunities and costs of occupation” [25]. But the prerequisite of sufficient capital for investment decisions has also not been met by all companies, which resulting in some companies not being able to truly represent their investment opportunities due to financing constraints [22, 26]. It’s pointed out that most SMEs with poor credit records and insufficient collateral present the above situation. Nevertheless, SMEs’ fiscal and financial policies can improve corporate financing constraints by enhancing their ability to raise capital, which can in turn have an incentive effect on corporate investment efficiency. Proactive fiscal policy can increase the supply of productive public capital to the relevant enterprises, and provide large amounts of direct or indirect financial support to enterprises in various forms, including subsidies, interest rate subsidies and guarantees [27]. The strength of enterprises’ own capital increases, and improvements in the expectations that commercial banks have of enterprises can also enhance their access to for credit. Financial incentives make it easier for SMEs to obtain investment funds through measures that include developing small and medium-sized financial institutions, SME credit departments of large commercial banks, regional equity markets and supporting high-quality SMEs to go on the market. Through policy measures that include improving the credit environment and the credit guarantee system, SMEs can obtain investment funds at a lower cost and reduce the cost of capital. A large number of studies have been conducted that prove the effectiveness of these policy measures in alleviating financing constraints and enhancing the financing ability of SMEs [28, 29].

This paper draws on the preceding analysis to argue that SMEs’ fiscal and financial policies affect SMEs’ investment efficiency through two paths: investment opportunities and financing capacity. On this basis, it proposes its first hypothesis:

*Hypothesis 1*: *SMEs’ fiscal and financial policies have an incentive effect on their investment efficiency*.

However, there are also differences in the nature of property rights among SMEs as, when compared to state-owned enterprises (SOEs), non-SOEs have poorer financing capacity, fewer available investment opportunities and a weaker ability to grasp quality investment opportunities. Chinese financial institutions discriminate against non-SOEs in credit rationing [30], and credit resources are preferentially allocated to SOEs with close ties and guarantee to the government [31]. A side proof is that China’s anticorruption can mitigate above mentioned distortions in the credit market [32]. In terms of investment opportunities, the natural ties between SOEs and the government help them break through industry barriers and enter monopolistic and regulated industries with high profit margins such as energy and transportation. Contrariwise, non-SOEs are discriminated in industry access and investment project approval, which further widens the gap between SOEs and non-SOEs in investment opportunities. What’s more, when there are good investment opportunities, SOEs are better-placed than non-SOEs to grasp investment opportunities and this is because SOEs face mild financial constraints, whereas non-SMEs experience severe financial constraints. The heterogeneity of property rights affects SMEs’ fiscal and financial policies in two ways:

On the one hand, we would like to see state-owned SMEs, which have financing capacity and investment opportunity advantages over non-state-owned SMEs, weaken the incentive effect of fiscal and financial policies on their investment efficiency. This would make non-state-owned SMEs, with weaker financing capacity and investment opportunities, to be more sensitive to fiscal and financial support policies, which would in turn make their investment efficiency more obviously incentivized. On this basis, we propose the following hypothesis:

*Hypothesis 2A*: *Relative to state-owned SMEs*, *fiscal and financial policies have a more significant incentive effect on the improvement of the investment efficiency of non-state-owned SMEs*.

On the other hand, we take into account that even with the support of fiscal and financial policies, non-SOEs probably still suffer explicit and implicit system discrimination from the credit and capital markets. The “advocating the rich and hating the poor” and “seeking profits and avoiding risks” lending idea of commercial banks and the “credit discrimination” against non-SOEs cannot be completely resolved in the short term [33, 34]. With regard to investment opportunities, it has been found that enterprises with more investment opportunities are more likely to receive credit financing compared to enterprises with fewer investment opportunities when there is loose monetary policy [35]. The implementation of fiscal and financial policies may therefore cause more credit resources and investment opportunities to flock to SOEs, which is something that policy makers do not want to see, but may be more consistent with the reality in China. We propose the following hypothesis:

*Hypothesis 2B*: *Relative to non-state-owned SMEs*, *fiscal and financial policies have a more significant incentive effect on improvements in the investment efficiency of state-owned SMEs*.

In addition, the size of an enterprise also has an important impact on its ability to raise capital and therefore on investment opportunities. Jin [36] empirically measured the extent of capital mismatch due to “scale discrimination” and showed that “scale discrimination” is twice of “ownership discrimination” in both relative and absolute terms. In referring to SMEs, “scale discrimination” was found to be the main source of external constraints on the financing structure of SMEs, rather than “ownership discrimination” [37]. And within the group of SMEs, “scale discrimination” between medium-sized enterprises and small enterprises still exists. Cheng & Ding [38] showed that in the case of SMEs, the probability of obtaining bank loans increases by 1.58 percent for every 10 employees; 3.78 percent for every 100,000 RMB increase in fixed assets; and 2.33 percent for every 100,000 RMB increase in main business income. When the enterprise is smaller in size, there are less credit resources available. And even with policy support, the situation for small-scale enterprises does not seem to be as good as it should be. Even with the support of loose monetary policy, it is still the larger enterprises that can get more loans from banks, and loans have a limited impact on smaller enterprises [39].

And with strong financing constraints and low financing capacity, small enterprises naturally have a poor grasp of investment opportunities. As in the heterogeneity of property rights nature, there are also two cases of heterogeneity in SMEs’ fiscal and financial policies enterprise size:

*Hypothesis 3A*: *Relative to medium-sized enterprises*, *fiscal and financial policies have a more significant incentive effect on improvements in small enterprises’ investment efficiency*.
*Hypothesis 3B*: *Relative to small-sized enterprises*, *fiscal and financial policies have a more significant incentive effect on improvements in medium-sized enterprises’ investment efficiency*.

## Construction and analysis of SMEs’ fiscal and financial policy index

### Quantitative guidelines for SMEs’ fiscal and financial policy

In order to estimate the effect of fiscal and financial policy, we should first be able to measure fiscal and financial policy changes (i.e., to describe fiscal and financial policies in quantitative terms). In previous studies, industrial policies and SME supportive policies at the macro level usually appear in a regression equation, where they take the form of dummy variables expressed as explanatory variables (e.g., [19]). However, the problem is that policy intensity is not accurately reflected. Sun [40, 41] gave us a clear example by applying the narrative approach that Friedman and Schwartz used in their Monetary History of the United States [42] to identify policy intentions. By summing up the indices of individual instruments, she builds a time series of an overall indicator that she then uses to measure macroprudential ease/tightness. In this paper, our identification strategy uses quantitative and narrative approaches to study the Chinese central government’s policy documents. We collect and read in detail 517 SME financial and fiscal policies issued by more than 20 institutions Including the Central Committee of the Communist Party of China (CPC Central Committee); the State Council (including the General Office), the National People’s Congress(NPC); the Ministries of the State Council (National Development and Reform Commission; Ministry of Finance; the People’s Bank of China; Ministry of Commerce; Ministry of Industry and Information Technology; Ministry of Science and Technology; Ministry of Agriculture; Agencies Directly under the State Council (State Taxation Administration, State Administration for Market Regulation); and Institutions directly under the State Council (Banking and Insurance Regulatory Commission, Securities Regulatory Commission) (both independently and in collaboration) in the period 2000–2020.

In order to better reflect the policy’s content validity, more accurately study the policy variables themselves and construct the policy index, we mainly draw on Peng et al [43] to refine the policy itself in the two dimensions of policy strength and policy measures. The score of policy strength is based on the structure of state administrative power (see S1 Appendix for further details) and the type of policy and the types of SMEs’ fiscal and financial policies issued by the state and ministries that this paper collects. We focus on the main issuing departments of fiscal and financial support policies, and refer to the *Regulations on the Handling of Official Documents by Party and Government Organs (No*. *14 [2012])* issued by the General Office of the CPC Central Committee and the General Office of the State Council. This enables us to determine criteria for assigning the strength of SMEs’ fiscal, tax and financial policies (see Table 1).

**Table 1:** Criteria for assigning value to policy strength.

Score	Policy strength
5	Laws of the NPC and its Standing Committee; Opinions of the Central Committee of CPC and the State Council; regulations of the State Council
4	Opinions of the General Office of the Central Committee of CPC and the State Council; orders, opinions, measures and interim provisions of NDRC, Ministry of Finance, the People’s Bank of China, State Taxation Administration, Banking and Insurance Regulatory Commission, Securities Regulatory Commission
3	Opinions, measures and interim provisions of other Ministries, Directly-affiliated agencies and public institutions under the State Council
2	Circular of (General Office of) the State Council; Notices and announcements of NDRC, Ministry of Finance, the People’s Bank of China, State Taxation Administration, Banking and Insurance Regulatory Commission, Securities Regulatory Commission
1	Notices of other Ministries, Directly-affiliated agencies and public institutions under the State Council

The scope of SMEs’ fiscal and financial policies that this paper refers to include fiscal expenditure policies, tax policies and financial policies that are used to support SME development.

The fiscal expenditure policies that support the development of SMEs are divided into direct support mechanism and indirect support mechanism in accordance with their modes of action. The direct support mechanism directly applies financial expenditure to SMEs, including setting up special funds for SMEs and special financial funds; the indirect support mechanism seeks to apply financial expenditure to SMEs’ intermediary service institutions, including various service platforms, bases and guarantee institutions. In using the direct support mechanism, SMEs can directly obtain various kinds of subsidies and subsidies for their development and alleviate their financial difficulties; meanwhile, the indirect support mechanism enables the development environment of SMEs to be improved, and to in turn benefit from improved service capacity and intermediaries’ business expansion.

Although the tax policy that supports SME development belongs to the same category of fiscal policy as fiscal expenditure policy; meanwhile, the tax policy has different roles and measures from the fiscal expenditure policy, and its policy can be a self-contained system that are directly (such as one or two kinds of taxes exempted or halved for SMEs within a certain period) and indirectly For example, tax benefits that are applied to institutions and seek to support the development of SMEs–these include one-two kinds of taxes exempted for SMEs credit guarantee institutions and SME’s public services, financing and other platforms within a certain period provided to SMEs.

**Fig 1:**
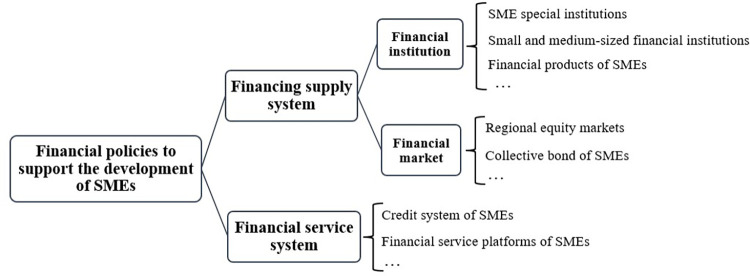
Framework of financial policies that support the development of SMEs. (See S2 Appendix for further details).

The financial policy that supports the development of SMEs is an important launching point to solve current difficulties in financing and meeting the high comprehensive cost of financing for SMEs. We divide SMEs’ financial policies into policies that support the financing supply system for SMEs, and policies that support the financial service system for SMEs. The external financing of enterprises is divided into direct financing and indirect financing which is the main external financing option for SMEs [44]. The development of indirect financing for SMEs requires financial institutions as the main policy target. SMEs are still mainly financed through bank loans [25], and so guiding banks to tilt their lending business to them is a top priority. Specific measures include the establishment of special institutions for SMEs; the development of small and medium-sized financial institutions; and the production of innovative financial products. The establishment of a perfect market financing system for SMEs is the highest realm of financing for SMEs. The main measures used to establish multi-level capital market financing include supporting high-quality SMEs to enter the market and financing, promoting innovative pilot regional equity markets and developing a collective bond between SMEs. In addition to providing policy support at the level of SMEs’ financing supply system, it is also necessary to improve the financial service system with the aim of alleviating the financing difficulties and expensive financing of SMEs. For example, a sound credit system of SMEs can weaken their financing barriers and improve their credit rating level, while sharing the loan risks of financial institutions. All SME financial service platforms can build a “one-stop” service platform that helps to sustain enterprise financing activities. Fig 1 provides a concise framework for SMEs’ financial policies.

After analyzing the fiscal expenditure, taxation and financial policies, we refined their policy measures and established criteria for assigning fiscal and financial policy measures to SMEs, as shown in Table 2.

**Table 2:** Criteria for assigning value to policy measures. (See S3 Appendix for further details).

**Score**	**Fiscal spending policy measures**
5	The central government set up special funds for SMEs. The amount is very clear, the regulations on the use of funds are detailed. The strength of policy measures is sat at the strongest level.
4	The central government sets up special funds to provide funds for SMEs in specific industries and fields, or for SME service institutions (referring to such as various service platforms for SMEs, small and micro enterprise innovation and entrepreneurship base, and SME credit guarantee institutions).The amount is very clear, the regulations on the use of funds are detailed. The strength of policy measures is relatively strong.
3	Local governments set up special funds for SMEs, or for SMEs in specific industries or fields, and provide financial support for SME service institutions. But the amount is not clear, the regulations are less detailed. The strength of policy measures is relatively strong.
2	Financial support in government procurement, policy subsidies, awards, loan subsidies to SMEs, relevant institutions that support SME development (such as various SME service institutions and financial institutions that provide financial support for SMEs and enter into financial contracts with SMEs). The strength of the policy measures is relatively general.
1	Involves the management of SME funds, the amount and use of funds. There is mention of strengthening SME-related funds to support specific industries, fields, and related institutions, as well as improving related fiscal policies. But detailed regulations and clear amounts are not given.
**Score**	**Tax Policy Measures**
5	Give SMEs themselves great tax incentives, exempting or halving 1~2 kinds of taxes for a certain period of time. The strength of policy measures is the strongest.
4	Give credit guarantee institutions for SMEs, various service platforms for SMEs, financial institutions that provide financial support for SMEs and sign financial contracts with SMEs great tax benefits, exempt from several taxes for a certain period of time, and allow pre-tax deductions for a larger portion of the amount; give SMEs themselves a certain degree of tax benefits, such as providing a lower tax rate and pre-tax deductions of less than 50% of the amount.
3	Give SMEs in specific industries and fields, industrial bases which take SMEs as the main service objects larger tax incentives, exempt from several taxes for a certain period of time, allowing pre-tax deduction of part of the amount.
2	Give taxpayers who invest in SMEs or donate to SME-related funds a certain level of tax incentives. The strength of the policy measures is relatively general.
1	Involves the implementation of the introduced tax incentives for SMEs. There is mention of continuing to formulate tax policies as needed, but no specific measures.
**Score**	**Financial Policy Measures**
5	Increase the support of financial institutions credit funds to SMEs. Set targets for the scale and growth rate of SME loans. The policies are straightforward and the measures are detailed. The strength of policy measures is the strongest.
4	Accelerate the pace of commercial banks and insurance companies to set up specialized institutions for financial services for SMEs. Form differentiated and characteristic incentive and restraint mechanisms within financial institutions. Vigorously carry out financial innovation, develop financial products adapted to the development of SMEs, and innovate service methods. Accelerate the development of small and medium-sized financial institutions for small (micro) enterprises.
3	Make full use of financing function of the multi-level capital market, broaden the financing channels of SMEs. Clearly put forward measures to support high-quality SMEs to go on the market and financing, construct the regional equity markets, develop the collective bonds of SMEs, play the role of financial leasing, trust and other financing measures.
2	Promote the construction of various financial service platforms for SMEs; establish and improve the credit guarantee system for SMEs. Promote the construction of credit system for SMEs. Policy measures are indirect, and the strength of it relatively general.
1	It is mentioned that financial policies suitable for the development of SMEs should be formulated to increase credit support for SMEs and broaden their financing channels, but there are no specific and detailed regulations. Improve the efficiency of financial services for SMEs, such as decentralizing loan approval authority, improving the efficiency of loan approval, and opening green channels. Granting SMEs deferment of capital and interest repayment arrangements.

### The calculation method of SMEs’ fiscal and financial policy index

In drawing on the aforementioned criteria for assigning the strength and measures of fiscal and financial policies for SMEs, we construct the SME fiscal and financial policy index by referring to the following calculation formula:

$${Index}_{i,t}=P_{i,t}*(E_{i,t}+T_{i,t}+F_{i,t})$$ (1)

In specification (1), *i* represents each SMEs’ fiscal and financial policy; *t* represents the time of policy introduction; *P*_*i*,*t*_ represents the policy strength (Power) score of policy *I*; *P*_*i*,*t*_ = {x∈N|1≤x≤5}; *E*_*i*,*t*_ represents the fiscal expenditure policy measure (Expand) score of policy *i; T*_*i*,*t*_ represents the tax policy measure (Tax) score of policy *I*; *F*_*i*,*t*_ represents the financial policy measure (Finance) score of policy *I*; and *E*_*i*,*t*_ = *T*_*i*,*t*_ = *F*_*i*,*t*_ = {x∈N|1≤x≤10}.For each policy, the scores of each type of measure are summed, and *Index*_*i*,*t*_ is the final quantitative value of SMEs’ fiscal and financial policies for policy *i* at time *t*. We summarize the issuance time of 517 policies by month and sum up quarterly to form this quarterly data of the SMEs’ fiscal and financial policy index. Although subjectivity is inevitable in the process of constructing the fiscal and financial policy index, we strive for reasonable and scientific quantitative criteria. The paper’s policy strength and measure assignment criteria were developed after a close reading of 517 policies, and the scoring was done after a second close reading. In the next step, we plot the trend of the SMEs’ fiscal and financial policy index, and focus on the peaks of the higher SMEs’ fiscal and financial policy index; we then verify the rationality of the SMEs’ fiscal and financial policy index by undertaking an analysis of the economic development situation and the production and operation of SMEs.

### Trend analysis of SMEs’ fiscal and financial policy index

**Fig 2:**
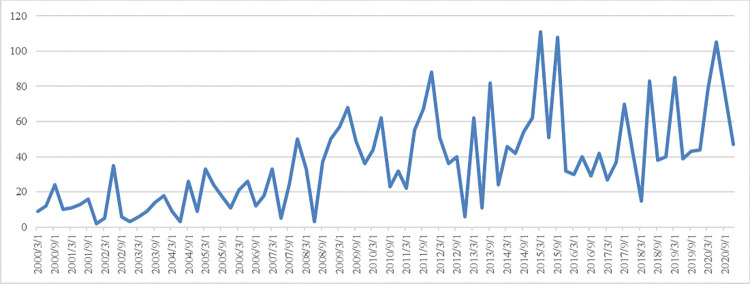
Trend of SME fiscal and financial policy index.

Fig 2 presents the trend of SME fiscal and financial policy index. In 2000, China’s accession to the WTO created opportunities and challenges for the development of SMEs, and various supportive policies were introduced that began to improve SME development. In the period between the end of 2008 and the middle of 2009, the international financial crisis caused changes in the domestic and international economic situation. SMEs are in a disadvantaged position in the market economy, and so they were inevitably the most affected group, and experienced greater production and operation difficulties and even faced the risk of closure and collapse. In seeking to improve the production and business environment of SMEs, the Chinese government promptly introduced fiscal and financial policies and measures to support them. Although the production and operation of SMEs improved in the first half of 2009, there were continued financing and guarantee difficulties. In seeking to further consolidate and expand gains made in response to the financial crisis, and as China’s economic growth rate began to decline, support for SMEs further increased in 2011. In particular, “micro-enterprises” were classified for the first time by the *Notice on the Issuance of the Standard Regulations for the Classification of Small and Medium Enterprises*, which was issued in June, and fiscal and financial support for small and micro enterprises also intensified. In 2012, the convening of the 18th CPC National Congress led to the gradual improvement of the policy environment for SMEs and further increases in fiscal and financial support. In 2014, when the “new normal” of China’s economy was first proposed, China’s economic growth rate fell by 3 percentage points (compared to 2010) and the real interest rate rose by 3.76 percentage points, a significant deviation from the two trends that increased the burden of corporate financing costs. This is inseparable from the macroeconomic policy mix of “active fiscal policy + prudent monetary policy” that has been in place since 2011. This will further push up interest rates, “making it more difficult and expensive for SMEs to raise funds” [25]. In this situation, it is important to solve the financing problem of small (micro) enterprises, as this will help to stabilize growth, promote reform, adjust the structure and benefit people’s livelihood. In September 2015, the 104th State Council executive meeting decided to establish a national SME development fund with a total scale of 60 billion yuan, promote the establishment of credit risk compensation funds and re-guarantee funds and credit guarantee insurance funds for small and micro enterprises by all levels of government. Fiscal spending policies have been significantly strengthened. In 2020, COVID-19 had a major impact on macroeconomic operations, and a large number of SMEs faced a short-term survival crisis: manufacturing SMEs faced a serious shortage of orders, and service SMEs completely shutdown, leading to an increased risk of capital chain breakage and difficulties in resuming work and production. In order to help SMEs to tide over the difficulties, the central government used financial subsidies, tax refunds and reductions, financing guarantees and other fiscal and financial support policies.

## Research design: Sample, variables, descriptive statistics

### Samples

In this paper, we select A-share enterprises listed on the Shenzhen and Shanghai Stock Exchange in the period 2000–20, classify SMEs based on their 2020 annual report financial data in accordance with *the Statistical Classification of Large*, *Small*, *Medium and Micro Enterprises (2017)* (National Statistics [2017] No. 213), remove the financial category and ST enterprises, and collect a total of 967 SMEs of unbalanced panel data. Since the number of listed SMEs is already small, and the advantage of unbalanced panel data is precisely the larger sample size, which can maximize the use of the information contained in the data and can greatly alleviate the problem of sample selection bias caused by the small sample size of balanced panel data.

All data used in this paper are derived from the Wind database—in order to eliminate the effect of extreme values, the 1 percent and 99 percent percentile of the continuous variables for enterprises are processed. Winsorize processing and the following descriptive statistics and empirical results are based on the results of the processed data.

### Variables

#### Explained variable

In referring to Jin et al. [21], we use the sensitivity between investment expenditure (*Investment*) which standardize with total assets and profitability (*ROE*) as an indicator to examine investment efficiency. The performance is the coefficient of the interaction term of profitability (*ROE*) and SMEs’ fiscal and financial policy index (*Ln(index)*)–if the coefficient of interaction term is positively significant, it indicates that the sensitivity between investment and profitability is stronger and the investment efficiency is improved.

#### Explanatory variable

The main explanatory variable in this paper is the SMEs’ fiscal and financial policy index (*Ln(index)*) that is based on 517 policies and expressed as a logarithm. In order to examine the effect of the special fiscal and financial policies on small and micro enterprises, and more specifically their alleviation of the financing constraints of small businesses after the introduction of the concept of "small and micro" enterprises, we also construct an index of fiscal and financial policies for small and micro enterprises (*Ln(micro)*)–this is based on 215 fiscal and financial policies for small and micro enterprises from the period September 2011-December 2020, and is also expressed as a logarithm.

#### Control variables

In this paper, we select the factors that influence the amount and efficiency of investment by SMEs and assess them from both micro and macro perspectives. In engaging at the micro level of firms, and referring to most of the corporate finance literature, we control four variables: debt to asset ratio (*Lev*) which measures the level of indebtedness and the degree of risk; the growth indicator, operating income growth rate (*Growth*); the profitability indicator, operating profit ratio (*Profit*), and the cash flow (*CFO*)and cash holding (*Cash*). In engaging at the macro level, we use the real *GDP* and *M2* growth rates to control for economic conditions and monetary policy, and take into consideration the fact that changes in the macroeconomy and adjustments in monetary policy affect the credit supply of financial institutions as well as the financial position and external financing capacity of enterprises. Moreover, entrepreneurs’ perceptions and confidence in the macroeconomic environment also impact enterprises’ investment behavior, and so the confidence index of medium and small entrepreneurs (*Confidence1*, *Confidence2*) are also controlled separately.

#### Variables in conduction mechanism model

In order to examine the mechanism of investment opportunity, this paper aligns itself with most of the literature to use Tobin’s Q as a proxy variable for investment opportunity [45]. In examining the mechanism of financing capacity, it draws on Almeida et al. [46] and introduces variables such as change in cash holdings (*DCash*), enterprise size [*Ln(Size)*], change in short-term debt (*DStd*), change in net working capital (*DNwc*) and capital expenditure (*Expand*), which it uses to measure the financing constraints of SMEs by building the cash-cash flow sensitivity model.

### Descriptive statistics

Table 3 shows the descriptive statistics of the aforementioned main variables: the mean value of enterprise investment is 0.0337, and the difference between the maximum and minimum values is 0.2408, which reflects some variability. The log mean of SMEs’ fiscal and financial policy index is 3.9201 with a standard deviation of 1.1823, which indicates a large variation in policy intensity, compared to the log mean of the fiscal and financial policy index for small (micro) enterprises of 4.0808 with a standard deviation of 0.8558. This indicates that, in overall terms, the fiscal and financial policy intensity for small enterprises is greater and more stable. The speculative opportunity variable *Tobin’s Q*, which reflects enterprises’ growth capability, also shows a large difference. With regard to control variables, the mean value of SME operating income growth rate is 0.185 –this is low relative to the country’s main board listed enterprises, and indicates that SMEs have low growth levels and more room for growth. Enterprises’ average debt to asset ratio is 37.8 percent, which indicates that, on average, SMEs have a slightly lower level of debt financing.

**Table 3:** Descriptive statistics.

	OBS	MEAN	STD	MIN	MAX
*Investment*	29118	0.0300	0.0312	0.0002	0.1811
*Ln(index)*	80572	3.9201	1.1823	0.6931	5.6454
*Ln(micro)*	36442	4.0808	0.8558	1.7918	5.3423
*ROE*	26051	0.0576	0.1162	-0.4660	0.4552
*Lev*	19450	0.3523	0.2322	0.3362	1.1499
*CFO*	25076	0.0122	0.0794	-0.2735	0.3485
*Cash*	24193	0.2211	0.2523	0.0029	1.633
*Growth*	27960	0.1697	0.6112	-0.8153	4.5967
*Profit*	31640	0.0008	0.0033	-0.0200	0.0097
*Confidence1*	72884	4.8070	0.0734	4.5257	4.9251
*Confidence2*	72884	4.7376	0.0668	4.4942	4.8331
*M2*	80572	0.0356	0.0181	-0.0062	0.1167
*GDP*	79611	0.0241	0.0206	-0.0983	0.1288
*LogTQ*	23349	0.8724	0.8259	-1.5731	3.0380
*DCash*	27746	0.0077	0.1196	-0.3126	0.4980
*Ln(Assert)*	28111	20.7042	0.8948	18.2507	22.9140
*DStd*	27948	0.2933	0.1962	0.0215	0.9569
*DNwc*	15486	0.0068	0.0974	-0.3924	0.5969
*Expand*	22373	0.0317	0.0453	0	0.2530
*Ln (USepu)*	80572	4.6789	0.4246	3.9196	6.1126
*Inveff*	15096	0.0113	0.1595	0	0.2646

## Empirical research: Model and results

### Estimation of the fiscal and financial policy efficacy

#### Baseline analysis

In order to explore the impact of SMEs’ fiscal and financial policies on investment efficiency, this paper constructs the following specification by referring to Jin et al. [21].


$${\mathrm{Investment}}_{i,t}=\alpha_{0}+\alpha_{1}{Ln(\mathrm{index})}_{t}+\alpha_{2}{ROE}_{i,t‐1}+\alpha_{3}{\mathrm{Ln}(\mathrm{index})}_{t}*{ROE}_{i,t‐1}+\alpha_{4}{\mathrm{Lev}}_{i,t-1}+\alpha_{5}{\mathrm{Growth}}_{i,t}+\alpha_{6}{\mathrm{CFO}}_{i,t}+\alpha_{7}{\mathrm{Cash}}_{i,t}+\alpha_{8}{\mathrm{Growth}}_{i,t}+\alpha_{9}{\mathrm{Profit}}_{i,t}+\alpha_{10}{\mathrm{Confidence1}}_{i,t}+\alpha_{11}{\mathrm{Confidence}2}_{i,t}+\alpha_{12}{M2}_{i,t}+\alpha_{13}{\mathrm{GDP}}_{i,t}+\varepsilon_{i,t}$$ (2)

The investment efficiency variable is examined using the sensitivity between investment expenditure (*Investment*) and profitability (*ROE*), and the coefficient of interaction term *α*_3_ of *ROE* and policy index (*Ln(index)*) is the main test variable. On the basis of Hypothesis 1, we expect the coefficient of *α*_3_ to be significantly greater than zero (i.e., fiscal and financial policies will have a significant incentive effect on the investment efficiency of SMEs). For all following regressions, fixed effects regression models that control for individuals and provinces are used and time fixed effects are not controlled for. Because the paper’s policy index (*Ln(index)*) is a macro variable in the time series, controlling for time fixed effects would produce perfect collinearity and lead to nonsense parameters. The baseline regression results are reported in Table 4, where column (1) does not include any control variables, and the coefficient of the interaction term Ln(index)*ROE is significantly positive at the 1% significance level, indicating that fiscal and financial policies enhance the sensitivity between SME investment and profitability, and suggest investment efficiency is positively incentivized. Columns (2) and (3) incorporates firm-level and macro-level control variables respectively; and Columns (4) controls for both micro and macro factors. As can be seen, the coefficient of interaction term between investment level and *ROE* remains significantly positive after the inclusion of the control variables, and there is no significant change in the significance and coefficient. This is consistent with our expectation, and Hypothesis 1 is supported.

**Table 4:** Empirical results of SMEs’ fiscal and financial policies and investment efficiency.

	*Investment*			
	(1)	(2)	(3)	(4)
*ROE*	-0.0319** (-2.24)	-0.0452** (-2.06)	-0.0353** (-2.54)	-0.0394* (-1.88)
*Ln(index)*	-0.021*** (-9.26)	-0.0018*** (-5.55)	-0.0014*** (-6.12)	-0.0008** (-2.58)
ROE* Ln(index)	0.0088*** (3.08)	0.0102** (2.36)	0.0101*** (3.65)	0.0098** (2.40)
*Lev*		-0.0129*** (-3.47)		-0.0132*** (-3.81)
*CFO*		0.0432*** (7.72)		0.0318*** (6.39)
*Cash*		0.0032 (1.42)		0.0015 (0.70)
*Growth*		0.0006 (1.24)		0.0002 (0.39)
*Profit*		0.4657*** (3.11)		0.3272*** (2.17)
*Confidence1*			0.2434*** (20.96)	0.2005*** (14.72)
*Confidence2*			-0.2696*** (-22.52)	-0.2757*** (-21.10)
*M2*			-0.1460*** (-8.63)	-0.1328*** (-8.05)
*GDP*			0.0269*** (5.86)	0.2249*** (12.29)
*Firm Fixed Effect*	*Yes*	*Yes*	*Yes*	*Yes*
*Province Fixed Effect*	*Yes*	*Yes*	*Yes*	*Yes*
*Cons*	0.0355*** (33.07)	0.0378*** (18.75)	0.1441*** (9.49)	0.3774*** (13.86)
*Obs*	20379	14224	20379	14224
*R* ^*2*^	0.2932	0.2996	0.3392	0.3635

Notes: All results are OLS estimates; cluster-robust standard errors are in parentheses under coefficients; *, **, and *** represent 10%, 5%, and 1% significant levels, respectively.

#### Heterogeneity analysis

The heterogeneity analysis follows (the results are presented in Table 5). Consider first the results of the regressions that are grouped by the property rights nature of enterprise; the column (2) of the state-owned SMEs regression shows the coefficient of the interaction term Ln(index)* *ROE* is significantly positive at the 5% level, which is consistent with the baseline regression in column (1); meanwhile, the column (3) of the non-state-owned SMEs regression shows that the coefficient of the interaction term Ln(index)* *ROE* is insignificant, which indicates that the fiscal and financial policies on state-owned SMEs have stronger incentives for investment efficiency. Accordingly, Hypothesis 2B is supported.

**Table 5:** Heterogeneous empirical results of SMEs’ fiscal and financial policies and investment efficiency.

	*Investment*								
	(1)		(2)		(3)		(4)	(5)	
	Total	State-owned SMEs		Non-state-owned SMEs		Medium-sized enterprises			Small enterprises
*ROE*	-0.0394* (-1.88)	-0.0960* (-1.76)		-0.0182 (-1.02)		-0.0446* (-1.74)			-0.0110 (-0.47)
*Ln(index)*	-0.0008** (-2.58)	-0.0026*** (-3.74)		-0.0002 (-0.72)		-0.0008** (-2.42)			-0.0006 (-0.77)
ROE* Ln(index)	0.0098** (2.40)	0.0220** (2.01)		0.0053 (1.56)		0.0107** (2.16)			0.0053 (1.10)
*Lev*	-0.0132*** (-3.81)	-0.0133* (-1.67)		-0.0133*** (-3.50)		-0.0150*** (-3.83)			-0.0061 (-0.87)
*CFO*	0.0318*** (6.39)	0.0407*** (3.20)		0.0297*** (5.50)		0.0336*** (6.16)			0.0172 (1.58)
*Cash*	0.0015 (0.70)	-0.0067 (-0.92)		0.0029 (1.35)		0.0010 (0.46)			0.0039 (0.64)
*Growth*	0.0002 (0.39)	0.0002 (0.17)		0.0002 (0.33)		-0.0004 (-0.66)			0.0015 (1.67)
*Profit*	0.3272** (2.17)	0.3205 (1.14)		0.3408** (1.97)		0.6107*** (3.96)			-0.5041* (-1.78)
*Confidence1*	0.2005*** (14.72)	0.1332*** (5.71)		0.2232*** (13.70)		0.2061*** (13.91)			0.1481*** (4.43)
*Confidence2*	-0.2757*** (-21.10)	-0.2083*** (-8.70)		-0.2974*** (-19.63)		-0.2820*** (-20.19)			-0.2130*** (-5.89)
*M2*	-0.1328*** (-8.05)	-0.1234*** (-3.83)		-0.1347*** (-6.97)		-0.1382*** (-7.74)			-0.0925** (-2.26)
*GDP*	0.2249*** (12.29)	0.2154*** (5.51)		0.2208*** (10.39)		0.2228*** (11.38)			0.2331*** (4.13)
*Firm Fixed Effect*	*Yes*	*Yes*		*Yes*		*Yes*			*Yes*
*Province Fixed Effect*	*Yes*	*Yes*		*Yes*		*Yes*			*Yes*
*Cons*	0.3774*** (13.86)	0.3867*** (7.54)		0.3694*** (11.44)		0.3823*** (13.13)			0.3214*** (4.01)
*Obs*	14224	2994		11230		12752			1471
*R* ^*2*^	0.3635	0.2748		0.3807		0.3641			0.3576

Notes: All results are OLS estimates; cluster-robust standard errors are in parentheses under coefficients; *, **, and *** represent 10%, 5%, and 1% significant levels, respectively.

Turning to the results of the regressions grouped by enterprise size in columns (4) and (5) of the table, the differences between medium-sized and small enterprises are similar to those of state-owned and non-state-owned SMEs. The regression results of the medium-sized enterprise sample are basically consistent with the total sample, which shows fiscal and financial support policies have a facilitating effect on their investment efficiency. The coefficient of Ln(index)* *ROE* in the small enterprise sample is insignificant, indicating that fiscal and financial support policy has no significant incentive effect on the investment efficiency of small enterprises. Accordingly, Hypothesis 3B is supported.

Although non-state-owned and small enterprises are relatively disadvantaged in terms of investment opportunities and financing capacity, neither are more sensitive to fiscal and financial policies and get a greater boost in investment efficiency, as predicted by hypotheses 2A and 3A. This leads us to propose that while fiscal and financial policies can play an incentive role in the overall investment efficiency of SMEs, they mainly rely on the pull of the stronger state-owned and medium-sized enterprises. Non-state-owned and small enterprises do not fully enjoy the policy dividend, and are still in many respects caught in an “ownership” and “scale” discrimination dilemma. In the next step, we will study the conduction mechanism of fiscal and financial policies. By using the group-level regression of the conduction mechanism model, we can explore the reasons for the poor effect of supportive policies on non-state-owned and small enterprises.

China is currently in a transition period, and the capital market is not an efficient market. The special institutional background causes that macroeconomic policies have an important direct impact and guiding effect on the capital market and the investment behavior of enterprises. Previous macroeconomic policies mainly include economic cycle, fiscal policy, monetary policy, credit policy, tax policy, exchange rate policy, industrial policy and regulatory policy [47].

This paper’s baseline analysis reveals that SMEs’ fiscal and financial policies have an incentive effect on investment efficiency, and this verifies macroeconomic policy regulation that promotes the investment efficiency of micro enterprises in the SME category, and expands the research perspective of relevant literatures. Heterogeneity analysis shows macro-control is not as effective as imagined, and policy impact will intensify “discrimination”. Although this seems “cruel”, it is consistent with China’s reality. Some Chinese researchers offered evidence in support of property right nature heterogeneity when they observed non-state-owned enterprises improved investment efficiency through mixed-ownership reform, and the mechanism reduced financing constraints, which improved “discrimination” to a certain extent [48, 49]. In previous studies, size heterogeneity is usually reflected between large and medium enterprises, while the difference between medium and small (micro) enterprises is rarely discussed. A review of previous studies shows there are mainly two transmission paths that guide a country’s macroeconomic policy to impact the investment efficiency of micro enterprises: (1) Changes in enterprises’ overall judgment and expectations in relation to the national and industrial economy can be achieved through the guiding role of macroeconomic policies. They can then affect investment opportunities that influence enterprises’ investment behavior. (2) The influence of macroeconomic policies on interest rate and credit can cause changes in enterprises’ capital cost and financing ability, and this can in turn influence enterprises’ investment behavior.

We put forward that SMEs’ fiscal and financial policies play a positive role on investment efficiency through the two paths of investment opportunities and financing capacity. Not only test the existence of the two mechanisms, but also focus more on whether there is a difference between the nature of property rights and the scale of enterprises, which enables us to further explore the reasons why macro policies have such a poor support effect on non-state-owned and small enterprises.

### Estimation of the conduction mechanism model

#### Conduction mechanism analysis of investment opportunities

In order to test the existence of the path of investment opportunities, the following specification is constructed.


$${LogTQ}_{i,t}=\alpha_{0}+\alpha_{1}{Ln(\mathrm{index})}_{t‐1}+\alpha_{2}{\mathrm{Lev}}_{i,t‐1}+\alpha_{3}{\mathrm{Growth}}_{i,t}+\alpha_{4}{\mathrm{CFO}}_{i,t}+\alpha_{5}{\mathrm{Cash}}_{i,t}+\alpha_{6}{\mathrm{Growth}}_{i,t}+\alpha_{7}{\mathrm{Profit}}_{i,t}+\alpha_{8}{\mathrm{Confidence1}}_{i,t}+\alpha_{9}{\mathrm{Confidence}2}_{i,t}+\alpha_{10}{M2}_{i,t}+\alpha_{11}{\mathrm{GDP}}_{i,t}+\varepsilon_{i,t}$$ (3)

Where *LogTQ* represents the growth and investment opportunities of enterprises, the investment opportunities mechanism is presented in Table 6. Regardless of which samples is used, the results show a high significance of the coefficient of *Ln(index) (at the 1% level)*. This shows that SMEs’ fiscal and financial policies are positively related to investment opportunities, and supportive policies make a significant contribution to the improvement of SME investment opportunities. This confirms how fiscal and financial policies effect the investment efficiency of enterprises through investment opportunities. We also note that in the column (2), for the state-owned SMEs, the coefficient of *Ln(index)* is 0.0672, which is significantly lower than in the other samples. This may be due to the fact that, in the case of most SOEs, the investment environment and investment opportunities are already relatively better, and there is accordingly less scope for policy to improve their investment opportunities. It should also be concerned that the group-level results of the investment opportunity conduction mechanism do not explain the differences between state-owned and private, medium-sized and small enterprises that are reflected in the baseline regression. We will now test the mechanism of financing capacity.

**Table 6:** Empirical results of the investment opportunity mechanism.

	*Investment*				
	(1)	(2)	(3)	(4)	(5)
	Total	State-owned SMEs	Non-state-owned SMEs	Medium-sized enterprises	Small enterprises
*Ln(index)*	0.1106***	0.0672***	0.1322***	0.1016***	0.1527***
	(9.01)	(2.95)	(9.67)	(7.42)	(5.82)
*Lev*	-0.5417***	-0.5569**	-0.5254***	-0.5758***	-0.3402
	(-4.19)	(-2.13)	(-3.66)	(-4.02)	(-1.16)
*CFO*	-0.2360	-0.4292	-0.2025	-0.1928	-0.2942
	(-1.63)	(-1.33)	(-1.41)	(-1.22)	(-0.89)
*Cash*	0.8619***	1.4038***	0.7219***	0.8513***	0.9097***
	(8.69)	(6.85)	(8.39)	(7.88)	(4.54)
*Growth*	0.0436***	0.0363	0.0438**	0.0298	0.0760**
	(2.62)	(0.97)	(2.42)	(1.56)	(2.46)
*Profit*	0.9867	-6.3564	4.8945	1.4760	-0.4001
	(0.23)	(-0.66)	(1.27)	(0.28)	(-0.07)
*Confidence1*	0.3169	2.3849***	-0.3907	0.2779	0.6391
	(0.93)	(4.14)	(-0.93)	(0.73)	(0.83)
*Confidence2*	-1.4071***	-3.0117***	-0.8894***	-1.4831***	-0.9892
	(-4.71)	(-5.29)	(-2.60)	(-4.56)	(-1.27)
*M2*	-0.3899	1.1305	-0.8682	0.1095	-3.2878**
	(-0.77)	(1.20)	(-1.61)	(0.20)	(-2.40)
*GDP*	4.1734***	4.4130***	4.4256***	4.7348***	0.2594
	(7.20)	(4.16)	(6.41)	(7.57)	(0.16)
*Firm Fixed Effect*	*Yes*	*Yes*	*Yes*	*Yes*	*Yes*
*Province Fixed Effect*	*Yes*	*Yes*	*Yes*	*Yes*	*Yes*
*Cons*	5.4514***	3.0022***	6.3824***	6.0202***	1.8949
	(7.35)	(2.87)	(6.83)	(7.42)	(0.99)
*Obs*	16137	3845	12292	14256	1880
*R* ^*2*^	0.5427	0.5077	0.5515	0.5484	0.5219

Notes: All results are OLS estimates; cluster-robust standard errors are in parentheses under coefficients; *, **, and *** represent 10%, 5%, and 1% significant levels, respectively.

#### Conduction mechanism analysis of financing capacity

In order to test the path of “fiscal and financial policy-financing capacity-investment efficiency” of SMEs, we refer to Almeida et al. (2004) and Khurana et al. [50] to construct the following cash-cash flow sensitivity model:

$${\mathrm{DCash}}_{i,t}=\alpha_{0}+\alpha_{1}{CFO}_{i,t}+\alpha_{2}{CFO}_{i,t}*{Ln(\mathrm{index})}_{t‐1}+\alpha_{4}{\mathrm{Size}}_{i,t}+\alpha_{5}{DStd}_{i,t}+\alpha_{6}{\mathrm{DNwc}}_{i,t}+\alpha_{7}{\mathrm{Growth}}_{i,t}+\alpha_{8}{\mathrm{Expand}}_{i,t}+\varepsilon_{i,t}$$ (4)

*where*, *DCash* is the change in cash holdings of the enterprise; *CFO* is the firm cash flow; *Expend* is the enterprise capital expenditure; and *Growth* is the operating income growth rate. Its theory holds that if the enterprise has financing constraints, its cash flow sensitivity of cash holdings is positive, which is reflected in the fact that parameter *α*_1_ is significantly positive. *α*_2_ is the parameter of the interaction term between cash flow and fiscal and financial support policies- if *α*_2_ is significantly negative, it indicates that fiscal and financial support policies can significantly reduce the cash-cash flow sensitivity of SMEs and the financing constraint is effectively alleviated.

Table 7 shows that the cash flow *CFO* regression coefficient is significantly positive and is not affected by the property rights nature and size of enterprises, which indicates that, even with stock market financing access, listed SMEs still face external financing obstacles and exhibit significant cash-cash flow sensitivity. Of more interest to us is the coefficient of the interaction term CFO* Ln(index), which is significantly negative at the 5% level in the total sample. This indicates that, as a whole, fiscal and financial policies can significantly alleviate the financing constraints of SMEs, which effects the investment efficiency of enterprises by reducing financing constraints, demonstrating the existence of a mechanism of action of financing capacity. The group-level regression of property rights nature in columns (2) and (3) indicates that the coefficient of the interaction term CFO* Ln(index) is significantly negative of state-owned SMEs, but insignificant of non-state-owned SMEs, implying that the financing constraints of non-state-owned SMEs are not significantly alleviated with policy support. Similarly, the group-level regression of enterprise size in column (4) and (5) shows that the coefficient of the interaction term is significantly negative of medium-sized enterprises, but insignificant of small enterprises. So, the implementation of fiscal and financial policies also did not effectively reduce the cash-cash flow sensitivity of small enterprises. This may be caused by an actuality that of the SMEs supported by fiscal and financial policies, state-owned and medium-sized enterprises are able to obtain more credit resources and largely crowd out non-state-owned and small enterprises.

**Table 7:** Empirical results of the financing capacity mechanism.

	*Dcash*				
	(1)	(2)	(3)	(4)	(5)
	Total	State-owned SMEs	Non-state-owned SMEs	Medium-sized enterprise	Small enterprise
*CFO*	0.4846***	0.5610**	0.4700***	0.4487***	0.9131**
	(4.64)	(2.30)	(4.30)	(4.25)	(2.03)
CFO* Ln(index)	-0.0474**	-0.1153**	-0.0300	-0.0418**	-0.1225
	(-2.20)	(-2.20)	(-1.35)	(-1.97)	(-1.16)
*Ln(Assert)*	-0.0078**	-0.0043	-0.0087*	-0.0103**	0.0164*
	(-2.10)	(-0.80)	(-1.87)	(-2.57)	(1.83)
*DStd*	-0.0574***	-0.0086	-0.0763***	-0.0653***	-0.0172
	(-4.99)	(-0.60)	(-5.17)	(-5.17)	(-0.65)
*DNwc*	-0.0970***	-0.0780*	-0.1001***	-0.0986***	-0.0857*
	(-6.34)	(-1.96)	(-6.10)	(-6.21)	(-1.67)
*Growth*	0.0102***	0.0028	0.0132***	0.0140***	-0.0038
	(3.64)	(0.72)	(3.81)	(4.88)	(-0.60)
*Expand*	-0.1034**	0.0708	-0.1406***	-0.0962**	-0.2049
	(-2.42)	(0.82)	(-2.97)	(-2.17)	(-1.36)
*Firm Fixed Effect*	*Yes*	*Yes*	*Yes*	*Yes*	*Yes*
*Province Fixed Effect*	*Yes*	*Yes*	*Yes*	*Yes*	*Yes*
*Cons*	0.1835**	0.0886	0.2064**	0.2373***	-0.3287*
	(2.33)	(0.78)	(2.12)	(2.80)	(-1.79)
*Obs*	14358	2810	11548	13054	1304
*R* ^*2*^	0.1634	0.0979	0.1786	0.1569	0.2384

Notes: All results are OLS estimates; cluster-robust standard errors are in parentheses under coefficients; *, **, and *** represent 10%, 5%, and 1% significant levels, respectively.

Notably, the group-level regression n of the financing capacity mechanism reflects differences between state-owned and private, medium-sized and small enterprises that are consistent with those in the baseline regression. This suggests that financing capacity constrains the incentive effect of fiscal and financial policies on investment efficiency in the relatively disadvantaged group of private and small enterprises.

Many studies also reflect the determining role of financing capacity in SME investment efficiency [51, 52]. In China, Chen [53] studied high-growth SMEs that are mostly in sunrise industries or strategic emerging industries and found that they still face a strong financing difficulty dilemma. Liu & Wang [54] explored the impact of financing constraints and agency cost issues on SME investment efficiency, and found that the effect of financing constraints on SMEs is greater than agency costs—this presents the external manifestation of SME investment inefficiency as an underinvestment. This paper’s findings make it is clear that it is crucial to alleviate the financing constraints of private and small enterprises and help them to surmount “ownership” and “scale” discrimination in the financing process.

## Further analysis

### Estimation of the targeted policy efficacy

In our test of the financing capacity mechanism, we find that SMEs’ fiscal and financial policies do not significantly alleviate the financing constraints of small enterprises, and note this “weak point” obstacle impedes the efficiency of small firm investment. We speculate whether a wider range of SMEs’ supportive policies would not target small enterprises. To test this conjecture, we specifically extracted the fiscal and financial policies for small (micro) enterprises. The concept of “small and micro” enterprises was introduced to China in June 2011, and so we extracted 215 fiscal and financial policies specifically related to small and micro enterprises that were drawn from the period September 2011-December 2020. On this basis, we construct a cash-cash flow sensitivity model by using the fiscal and financial policy index of small (micro) enterprises *(Ln(micro))* and draw on a sample of 94 small enterprises. The regression results are shown in Table 8 in the form of a comparison with the fiscal and financial policy index *Ln(index)* of SMEs that relates to the period September 2011-December 2020. In accordance with our conjecture, the more targeted fiscal and financial policies for small (micro) enterprises have a significant mitigating effect on the financing constraints of small enterprises. The coefficient of CFO* Ln(micro) is significantly negative at the 5% level in column (1), while the fiscal and financial policies *Ln(index)* have no mitigating effect on the financing constraints of small (micro) enterprises, which is consistent with the results in the financing capacity mechanism.

**Table 8:** Empirical results of fiscal and financial policies for small (micro) enterprises to alleviate financing constraints.

	*Dcash*	
	(1)	(2)
	*Ln(micro)*	*Ln(index)*
*CFO*	1.1839*** (3.93)	0.9131** (2.03)
CFO* Ln(micro)	-0.1894** (-2.21)	
CFO* Ln(index)		-0.1225 (-1.16)
*Ln(Assert)*	0.0401*** (3.40)	0.0164* (1.83)
*DStd*	-0.0436 (-0.82)	-0.0172 (-0.65)
*DNwc*	-0.1367** (-2.30)	-0.0857* (-1.67)
*Expand*	-0.5401*** (-4.06)	-0.2049 (-1.36)
*Growth*	-0.0025 (-0.37)	-0.0038 (-0.60)
*Firm Fixed Effect*	*Yes*	*Yes*
*Province Fixed Effect*	*Yes*	*Yes*
*Cons*	-0.8087*** (-3.35)	-0.3287* (-1.79)
*Obs*	1025	1304
*R* ^*2*^	0.3322	0.1744

Notes: All results are OLS estimates; cluster-robust standard errors are in parentheses under coefficients; *, **, and *** represent 10%, 5%, and 1% significant levels, respectively.

### Robustness tests

#### Redefinition and measurement of explained variables

Another commonly accepted method for estimating the investment efficiency of enterprises is based on the Richardson [55] model. A group of representative variables that reflect the enterprise growth is first selected to establish the basic investment equation, i.e., specification (5). The expected investment level that is obtained through this model is considered to be the enterprise’s ideal investment level, and actual and expected investments are compared. The inconsistent part is the enterprise’s inefficient investment part.


$${\mathrm{Investment}}_{i,t}=\alpha_{0}+\alpha_{1}{\mathrm{Investment}}_{i,t‐1}+\alpha_{2}Log{TQ}_{i,t‐1}+\alpha_{3}{\mathrm{Lev}}_{i,t‐1}+\alpha_{4}{\mathrm{Size}}_{i,t‐1}+\alpha_{5}{\mathrm{Cash}}_{i,t‐1}+\alpha_{6}{\mathrm{Age}}_{i,t‐1}+\alpha_{7}{\mathrm{Return}}_{i,t‐1}+\mathrm{Year}+\varepsilon_{i,t}$$ (5)

In this equation, the control variables are all taken with a one-period lag, and the enterprise-quarter fixed effects model is used; two new variables (stock return (*Return*) and listed years (*Age*) are added. The residuals of specification (5) are considered as inefficient investment *(Inveff)*, which is used as the inverse measure of enterprise investment efficiency. The lagged period of SMEs’ fiscal and financial policy index *Ln(index)* is taken as the explanatory variable, and a fixed-effects model that controls for individual and province is constructed. The regression results are displayed in Table 9. Regardless of the inclusion of control variables, the coefficient of *Ln(index)* is always significantly negative at the 1% level.

**Table 9:** Robustness check: Replacing dependent variable.

	*Inveff*			
	(1)	(2)	(3)	(4)
*Ln(index)_*	-0.0011*** (-5.54)	-0.0010*** (-5.43)	-0.0009*** (-4.75)	-0.0009*** (-4.67)
*CFO*		0.0038 (1.29)		0.0037 (1.26)
*Growth*		0.0006 (1.53)		0.0005 (1.34)
*Profit*		0.1241 (1.61)		0.1353* (1.75)
*Confidence1*			0.0373*** (4.93)	0.0364*** (4.85)
*Confidence2*			-0.0418*** (-4.92)	-0.0413*** (-4.87)
*M2*			0.1065*** (7.98)	0.1070*** (8.07)
*GDP*			-0.0038	-0.0030
			(-0.68)	(-0.52)
*Firm Fixed Effect*	*Yes*	*Yes*	*Yes*	*Yes*
*Province Fixed Effect*	*Yes*	*Yes*	*Yes*	*Yes*
*Cons*	0.0160***	0.0157***	0.0318*	0.0331**
	(18.83)	(18.71)	(1.93)	(1.99)
*Obs*	14745	14721	14745	14721
*R* ^*2*^	0.0944	0.0958	0.1040	0.1054

Notes: All results are OLS estimates; cluster-robust standard errors are in parentheses under coefficients; *, **, and *** represent 10%, 5%, and 1% significant levels, respectively.

#### Discussion of endogeneity

Fiscal and financial policies are theoretically macro policies at the national level, while the micro behavior of individual enterprises hardly affects all macro policies. Therefore, there is almost no reverse causality between SME investment efficiency and fiscal and financial support policies. In empirical terms, this paper uses the U.S Economic Policy Uncertainty as an instrumental variable for SMEs’ fiscal and financial policies for the following reasons. The U.S EPU constructed by Baker et al. [56] is commonly used as an instrumental variable for the Chinese economic policy uncertainty indicator [57]. Economic uncertainty, in turn, is highly correlated with macroeconomic operations and a large number of supportive and subsidized policies. The central government uses them for the purpose of stabilizing the economy and reducing volatility; and local governments use them to increase performance pressure for promotion and extend “supporting hands” when economic uncertainty increases.

We report the above 2SLS estimations in Table 10. The results of the first stage show that regardless of which measurement for explained variables, the U.S. economic policy uncertainty *Ln (USepu)* is significantly positively correlated with the SMEs’ fiscal and financial policy index *Ln(index)* and ROE* Ln (USepu) also positively correlated with ROE*Ln(index) at the level of 1%. The Cragg-Donald F-statistics are much stronger than the standard threshold 10 suggested by Staiger and Stock [58], implying that our instruments are not weak. Then, the results of the second stage show that after controlling the potential endogeneity with instrumental variables, the effect of fiscal and financial support policies on SME investment efficiency is significantly positive at the 1% level.

**Table 10:** Robustness check: Instrumental variable replaces the explained variable.

*Second stage*	*Investment*			*Inveff*
	(1)			(2)
*Ln(index)*	0.0015 (0.67)			-0.0012*** (-2.73)
ROE* Ln(index)	0.0485*** (3.25)			
*ROE*	-0.2140*** (-3.07)			
*CFO*	0.0303*** (6.25)			0.0037 (1.27)
*Growth*	0.0000 (0.09)			0.0005 (1.27)
*Profit*	0.4260*** (3.04)			0.1376* (1.77)
*Confidence1*	0.2532*** (16.284)			0.0352*** (4.62)
*Confidence2*	-0.2679*** (-20.79)			-0.0407*** (-4.78)
*M2*	-0.1027*** (-5.56)			0.1051*** (7.69)
*GDP*	0.0260*** (3.77)			-0.0025 (-0.44)
*Firm Fixed Effect*	*Yes*			*Yes*
*Province Fixed Effect*	*Yes*			*Yes*
***First stage***	***Ln(index)***	ROE*Ln(index)		***Ln(index)***
*Ln (USepu)*	0.2851*** 42.63			0.7096*** (91.15)
ROE* Ln (USepu)			0.3957*** 7.07	

*Cragg-Donald F statistic*	329.374			2078.242
*Obs*	19743			14721
*R* ^*2*^	0.0531			0.0158

Notes: All results are 2SLS estimates; cluster-robust standard errors are in parentheses under coefficients; *, **, and *** represent 10%, 5%, and 1% significant levels, respectively.

#### Delete special samples

The 2008–2009 financial crise and the pandemics in 2020 caused great economic volatility and had a huge impact on SME operations. To increase the robustness of the paper’s findings, 12 quarters of sample data (from 2008, 2009 and 2020) were removed and the coefficient of the interaction term ROE* Ln(index) is found to be 0.0104 with a t-test of 2.35. This finding is significant at the 5% level and is consistent with the results of the baseline regression. Overall, the results from our robustness checks deliver a consistent message; i.e., SMEs’ fiscal and financial policies have an incentive effect on their investment efficiency, thus supporting Hypothesis 1.

## Conclusions and recommendations

This paper first uses quantitative and narrative approaches to identify and construct fiscal and financial policy index for SMEs that are based on SME supportive policies promulgated by the Central Government of China in the period 2000–20. It then examines the incentive effect and conduction mechanism of fiscal and financial policies on enterprise investment efficiency by drawing on a property rights nature perspective and referring to enterprise scale based on the financial data of listed SMEs in China. In doing so, it seeks to study the implementation of macro fiscal and financial policies from the micro perspective of enterprises. The relevant results follow:

➀ This paper finds that, on the whole, the SMEs’ fiscal and financial policies significantly enhance investment efficiency. However, the regression results grouped by the property rights nature of enterprise shows that the incentive effect of fiscal and financial policies on enterprise investment efficiency is stronger in state-owned SMEs and insignificant in private SMEs. The regression results grouped by enterprise size show that fiscal and financial policies can significantly enhance the investment efficiency of medium-sized enterprises, but are not significant in small enterprises.
➁ Investment opportunities and financing capacity are the two mechanisms through which SMEs’ fiscal and financial policies affect the investment efficiency.
➂ The group-level regressions of mechanism shows that there are differences in the transmission of mechanisms by property rights nature and enterprise size. The investment opportunities of state-owned SMEs are not significantly improved by policy support, and the financing capacity of private SMEs and small enterprises is not improved as much as state-owned and medium-sized enterprises. And the “ownership and scale discrimination” that private SMEs and small enterprises suffered in the financing process are key to limiting efficiency improvement.
➃ Further research shows that targeted fiscal and financial policies for small (micro) enterprises have a significant effect on alleviating the financing constraints of small enterprises. The following insights can be extracted from these findings:

First, this paper confirms that SMEs’ fiscal and financial policies have a certain incentive effect on the improvement of investment efficiency. In order to support SMEs, China has introduced a series of fiscal and financial policies, but they are phased, lack planning for long-term and overall development and are mostly general and not very operational. The central government should improve the top-level design, formulate more specific and detailed policies and measures, clarify standards that can be followed by local governments, and give SMEs the confidence to rise to the challenge and seek to improve themselves.

Second, gradually eliminate the (policy, finance and other) discrimination between private enterprises and state-owned enterprises, small enterprises and large and medium-sized enterprises. In order to enhance policy benefits for private, small and micro enterprises, the supportive policy for SMEs has shifted from “flood” to “precise drip” irrigation. This paper finds that there is less support (relief of financing constraints, access to credit resources, and policy incentives that enhance investment efficiency) for private and small enterprises than for state-owned and medium-sized enterprises. This is because private and small enterprises are in a disadvantaged position and are discriminated against in all aspects, making it difficult for them to enjoy/fully enjoy the SMEs’ policy dividend. The small (micro) enterprises as the main body that provides targeted fiscal and financial support policies can significantly improve the financing situation of small enterprises. This paper therefore suggests that the State Council and its Ministries that promulgate policies should provide targeted policy support to private SMEs and small (micro) enterprises, and give greater policy preference to private and small (micro) enterprises. These measures could include the establishment of a special fund for private SMEs; the establishment of a credit guarantee agency that specifically benefits private SMEs and small (micro) enterprises; and the establishment of a target for commercial banks that privileges loans to small (micro) enterprises over those provided to large and medium-sized enterprises.

In addition, we can consider the establishment of a management agency that specializes promoting the development of private and small (micro) enterprises on the basis of private and small (micro) enterprises. It should formulate unified policy measures for them, coordinate planning and promote the development of private and small (micro) enterprises.

Third, this paper finds that investment opportunities and financing capacity are the two pathways through which fiscal and financial policies work. The Government must therefore focus on enhancing investment opportunities for SMEs and alleviating financing constraints that inhibit SMEs. In seeking out investment opportunities, the Government should focus on private SMEs, relax industry barriers (so that private enterprises can freely enter all industries), eliminate differential treatment between state-owned and private enterprises and improve the efficiency and approval rate of private enterprises’ investment projects. This paper argues that, in order to reduce financial constraints and enhance credit support for SMEs, the scale and increase of SME loans should be included in the assessment index of commercial banks, and the scale and increase of loans to private SMEs and small (micro) enterprises should be included in the key assessment index of commercial banks. In order to enhance the role of large and medium-sized commercial banks in SME financing, the cultivation and vigorous development of a number of small and medium-sized financial institutions should also be accelerated. The central government should strengthen institutional support for SMEs, and local governments should develop differentiated and characteristic development paths for them, while also reducing intervention in the operation and management of SMEs and strengthening assistance that will enhance their independence.

Of course, this paper still has limitations: First, it uses a single data source for the explanatory variable (SMEs’ fiscal and financial policy index). The quantitative and narrative approaches that this paper uses to construct the SMEs’ policy index are relatively new, and so there is no similar index for comparison and reference. Although the trend chart of policy index conforms to the actual situation of China’s economy and society and the robustness test is conducted with the instrumental variable (Economic Policy Uncertainty of the U.S), there is still the possibility of error. Second, subject to data availability, the research object of this paper is China’s A-share listed SMEs. But the vast majority of SMEs are actually unlisted. This could affect the generalization of the paper’s conclusions.

We suggest that future research can be improved and expanded by engaging at the following two points:

First, construct a multi-level SMEs’ fiscal and financial policy index. This paper’s policy index only includes SMEs’ fiscal, tax and financial policies that were issued by the Central Government, and do not refer to counterpart policies issued by local governments of China. This recommendation cites two considerations. First, many local government supportive policies are specific implementations and forwarded versions of Central Government policies; Second, some early local government policies, especially at the city and county levels, have been invalidated or (as in the case of pre-2010 policies) cannot be obtained, which means the original policies are unobtainable. This can lead to omissions. We hope researchers will be able to balance the relationship between central and local government policies in the future, and thereby establish and improve the SMEs’ fiscal and financial policy index.

Second, the research sample of SMEs should be expanded and a wider range of research should be conducted. This is the biggest dilemma for SMEs. We hope that unlisted SMEs can enhance information disclosure and that special funds or subsidies will enable governments to disclose financial information related to SMEs. In the future, our team will also cooperate with local governments with the aim of obtaining more detailed and comprehensive micro data.

## Supporting information

S1 Appendix Explanation of Chinese power, administrative organs. (DOCX)

S2 Appendix Explanation of terms. (DOCX)

S3 Appendix Explanation of policy measures. (DOCX)

S1 Data (DTA)

S1 File (DO)
