# Supplementary material for: Can SMEs benefit equally from supportive policies in China?
Source: PLoS One. 2023 Mar 30;18(3):e0280253. doi: 10.1371/journal.pone.0280253 (PMC10062620; doi:10.1371/journal.pone.0280253)
Supplement: S1 Appendix — (DOCX) [file pone.0280253.s001.docx]

**S1 Appendix.**

**Explanation of Chinese power, administrative organs.**

| **Organ** | **Explanation 1** | **Explanation 2** |
| --- | --- | --- |
| The National People's Congress (NPC) | NPC is the highest organ of state power in China | The Constitution prescribes that the NPC and its Standing Committee exercise the legislative power of the state. |
| NPC Standing Committee | The NPC Standing Committee is a permanent organ of the NPC. |  |
| The Central Committee of the Communist Party of China | The Central Committee of (Communist Party of China) CPC is the core authority of the CPC. | The Central Committee of CPC is elected by the NPC. |
| The State Council | The State Council is the executive organ of the highest organ of state power and the highest state administrative organ. | The State Council has set up General Offices, Ministries (26), Ad hoc institution (1), Directly-affiliated agencies (16), Offices (2), Directly-affiliated public institutions (9), National bureaus managed by ministries and commissions (16). |
| Nation Development and Reform Commission (NDRC) | Ministries of the State Council | Build a policy coordination and work coordination mechanism for development planning, fiscal and financial matters. |
| Ministry of Finance | Ministries of the State Council | Coordinate fiscal and tax policies and regulations, and manage government investment funds. |
| The People's Bank of China | Ministries of the State Council | Formulation and adjustment of monetary policy, formulation and implementation of credit policy, supervision and structural adjustment of financial market |
| State Taxation Administration | Directly-affiliated agency under the State Council | Propose national tax policy recommendations and jointly review and report with the Ministry of Finance, formulate and implement measures. |
| Banking and Insurance Regulatory Commission | Directly-affiliated public institution under the State Council | Unified supervision and management of the banking and insurance industries. Formulate business rules and regulatory rules for other types of institutions such as small loan companies, financing guarantee companies, and financial leasing companies. |
| Securities Regulatory Commission | Directly-affiliated public institution under the State Council | Research and formulate the principles, policies and development plans of the securities and futures market; formulate regulations, rules and measures for the supervision of the securities and futures market. |
