# Supplementary material for: Can SMEs benefit equally from supportive policies in China?
Source: PLoS One. 2023 Mar 30;18(3):e0280253. doi: 10.1371/journal.pone.0280253 (PMC10062620; doi:10.1371/journal.pone.0280253)
Supplement: S2 Appendix — (DOCX) [file pone.0280253.s002.docx]

**S2 Appendix.**

**Explanation of terms.**

1. **Regional equity market:** China's capital market is divided into exchange market (main board, SME board, GEM board) and National SME stock system (New Third Board, Regional equity market). Regional equity market is a private equity market mainly serving micro, small and medium-sized enterprises in the provincial administrative region. It plays a positive role in promoting equity transaction and financing of micro, small and medium-sized enterprises, encouraging scientific and technological innovation and activating private capital, and strengthening support for weak links in the real economy.
2. **Collective bond of SMEs:** refers to the bond issued by an organization as the leader and several enterprises together, which belongs to a kind of corporate bond. Specifically, SMEs collective bond adopts the market operation modes of collective debt issuance, separate liabilities, unified guarantee, unified organization and is organized by the initiator who is responsible for selecting and monitoring the issuing bodies. Therefore, SMEs collective bond is a kind of “Agglomerating sand to pagoda” financing innovation, forming in practice in China. Some Chinese scholars have found that collective bonds can enable direct financing of SMEs which are difficult to issue bonds alone, breaking the ceiling of bond market financing and achieving a “0 to 1” leap.
3. **Service platform for SMEs:** refer to legal entities that provide regional and industrial SMEs with services such as information inquiry, technological innovation, quality testing, regulations and standards, management consulting, entrepreneurial guidance, market development, personnel training and equipment sharing in accordance with the principles of openness and resource sharing.
4. **Financial service platform for SMEs:** refer to a service platform focused on supporting the financing and development of micro, small and medium-sized enterprises. The platform usually uses big data, artificial intelligence, blockchain and other technical means to capture the effective information of micro, small and medium-sized enterprises, and match financial institutions with micro, small and medium-sized enterprises for investment and financing docking. In China, many provinces and cities have opened financial service platforms for small and medium-sized enterprises.
5. **Small and micro enterprise innovation and entrepreneurship base:** refers to all kinds of organizations and institutions that provide entrepreneurship and innovation places and services for small and micro enterprises. It includes all kinds of entrepreneurship bases, entrepreneurship parks, mass maker Spaces, incubators, economic and technological development zones, industrial parks, high-tech parks, and entrepreneurship and innovation bases (platforms) for small and micro enterprises, entrepreneurial teams, and makers set up by leading enterprises around their main business directions.
