# Supplementary material for: Can SMEs benefit equally from supportive policies in China?
Source: PLoS One. 2023 Mar 30;18(3):e0280253. doi: 10.1371/journal.pone.0280253 (PMC10062620; doi:10.1371/journal.pone.0280253)
Supplement: S3 Appendix — (DOCX) [file pone.0280253.s003.docx]

**S3 Appendix.**

**Explanation of policy measures.**

|  | **Measures** | **Explanation**  (Policies that have been implemented) |
| --- | --- | --- |
| **Fiscal spending policy** | The central government set up special funds for SMEs. | In 2010, the central government allocated 10.6 billion yuan in special funds to support the development of SMEs; In 2012, the total scale of special funds for SMEs was expanded from 12.87 billion yuan to 14.17 billion yuan; In 2015, a national SME development fund with a total scale of 60 billion yuan was established……. |
|  | The central government sets up special funds to provide funds for SMEs in specific industries and fields, or for SME service institutions (referring to such as various service platforms for SMEs, small and micro enterprise innovation and entrepreneurship base, and SME credit guarantee institutions) support. | In 2006, each year, 40 million yuan was invested in guiding funds, and local governments were guided to invest more than 200 million yuan to support the construction of various public service platforms for technology-based SMEs; In 2012, *Measures for the Institution of Credit Guarantee Funds for SMEs;* In 2018, the central government initiated and established the National Financing Guarantee Fund, with an initial fundraising of no less than 60 billion yuan to support small and micro enterprises…… |
| **Tax**  **Policy** | Give SMEs themselves great tax incentives, exempting or halving 1~2 kinds of taxes for a certain period of time. | In 2014, small and micro enterprises with monthly sales below 30,000 are exempt from business tax and value-added tax…… |
|  | Give credit guarantee institutions for SMEs, various service platforms for SMEs, financial institutions that provide financial support for SMEs and sign financial contracts with SMEs great tax benefits, exempt from several taxes for a certain period of time, and allow pre-tax deductions for a larger portion of the amount. | In 2007, eligible SME credit guarantee institutions were exempted from business tax; In 2012, the imported equipment of the National Public Service Demonstration Platform for SMEs (Technology) was exempted from import tax; In 2014, exemption of stamp duty on loan contracts signed between financial institutions and small and micro enterprises; In 2018, the interest income obtained by financial institutions from small loans to small enterprises and micro enterprises will be exempted from value-added tax.…… |
|  | Give taxpayers who invest in SMEs or donate to SME-related funds a certain level of tax incentives. | In 2007, venture capital companies that invest in unlisted high-tech SMEs can deduct taxable income based on a certain percentage of the investment amount……. |
| **Financial Policy** | Set targets for the scale and growth rate of SME loans. | In 2019, large state-owned commercial banks' loans to small and micro enterprises will increase by more than 30%, and the inclusive loans to small and micro enterprises will strive to achieve a year-on-year increase of more than 30% in the overall balance; In 2020, the growth rate of inclusive loans to small and micro enterprises by large commercial banks will be higher than 40%...... |
